# MYC promotes tryptophan uptake and metabolism by the kynurenine pathway in colon cancer

**DOI:** 10.1101/gad.327056.119

**Published:** 2019-09-01

**Authors:** Niranjan Venkateswaran, M. Carmen Lafita-Navarro, Yi-Heng Hao, Jessica A. Kilgore, Lizbeth Perez-Castro, Jonathan Braverman, Nofit Borenstein-Auerbach, Min Kim, Nicholas P. Lesner, Prashant Mishra, Thomas Brabletz, Jerry W. Shay, Ralph J. DeBerardinis, Noelle S. Williams, Omer H. Yilmaz, Maralice Conacci-Sorrell

**Affiliations:** 1 Department of Cell Biology, University of Texas Southwestern Medical Center, Dallas, Texas 75390, USA;; 2 Department of Biochemistry, University of Texas Southwestern Medical Center, Dallas, Texas 75390, USA;; 3 Koch Institute for Integrative Cancer Research, Department of Biology, Massachusetts Institute of Technology, Cambridge, Massachusetts 02139, USA;; 4 Lydia Hill Department of Bioinformatics, University of Texas Southwestern Medical Center, Dallas, Texas 75390, USA;; 5 Children's Medical Center Research Institute, University of Texas Southwestern Medical Center, Dallas, Texas 75390, USA;; 6 Department of Pediatrics, University of Texas Southwestern Medical Center, Dallas, Texas 75390, USA;; 7 Nikolaus-Fiebiger-Center for Molecular Medicine, University Erlangen-Nurnberg, Erlangen 91054, Germany;; 8 Harold C. Simmons Comprehensive Cancer Center, University of Texas Southwestern Medical Center, Dallas, Texas 75390, USA;; 9 Howard Hughes Medical Institute, Dallas, Texas 75390, USA;; 10 Department of Pathology, Massachusetts General Hospital Boston, Harvard Medical School, Boston, Massachusetts 02114, USA

**Keywords:** MYC, AHR, cancer, kynurenine, tryptophan metabolism, AFMID, SLC1A5, SLC7A5, organoid

## Abstract

Tumors display increased uptake and processing of nutrients to fulfill the demands of rapidly proliferating cancer cells. Seminal studies have shown that the proto-oncogene MYC promotes metabolic reprogramming by altering glutamine uptake and metabolism in cancer cells. How MYC regulates the metabolism of other amino acids in cancer is not fully understood. Using high-performance liquid chromatography (HPLC)-tandem mass spectrometry (LC-MS/MS), we found that MYC increased intracellular levels of tryptophan and tryptophan metabolites in the kynurenine pathway. MYC induced the expression of the tryptophan transporters SLC7A5 and SLC1A5 and the enzyme arylformamidase (AFMID), involved in the conversion of tryptophan into kynurenine. SLC7A5, SLC1A5, and AFMID were elevated in colon cancer cells and tissues, and kynurenine was significantly greater in tumor samples than in the respective adjacent normal tissue from patients with colon cancer. Compared with normal human colonic epithelial cells, colon cancer cells were more sensitive to the depletion of tryptophan. Blocking enzymes in the kynurenine pathway caused preferential death of established colon cancer cells and transformed colonic organoids. We found that only kynurenine and no other tryptophan metabolite promotes the nuclear translocation of the transcription factor aryl hydrocarbon receptor (AHR). Blocking the interaction between AHR and kynurenine with CH223191 reduced the proliferation of colon cancer cells. Therefore, we propose that limiting cellular kynurenine or its downstream targets could present a new strategy to reduce the proliferation of MYC-dependent cancer cells.

## Abstract (precis)

In this study, Venkateswaran et al. investigated how the proto-oncogene MYC regulates the metabolism of amino acids other than glutamine in cancer. They found that MYC increased intracellular levels of tryptophan and tryptophan metabolites in the kynurenine pathway and that blocking the kynurenine pathway caused preferential death of established colon cancer cells and transformed colonic organoids.

Tryptophan (Trp) is an essential amino acid for protein synthesis, and its metabolism can give rise to serotonin and metabolites in the kynurenine (Kyn) pathway (Palego et al. 2016). The Kyn pathway is mostly active in the liver, where it is responsible for metabolizing >90% of cellular Trp. Extrahepatically, the Kyn pathway is less active, with limited contribution to Trp degradation (Badawy 2017).

The Kyn pathway produces biologically active metabolites, including niacin (vitamin B_3_), picolinic acid, quinolinic acid, kynurenic acid, cinnabarinic acid, xanthurenic acid, and Kyn (Badawy 2017). The final product of the Kyn pathway is the redox cofactor NAD^+^ (Palego et al. 2016; Badawy 2017). Liver cells contain elevated levels of all of the enzymes necessary for complete metabolism of Trp to NAD^+^; however, the amounts and activity of different enzymes in the Kyn pathway in other tissues define the production rate and stability of specific Trp metabolites. Alterations in Trp metabolism correlate with the presence of neurological diseases, including depression and autism (Chen and Guillemin 2009; Zhou et al. 2018a,b) and cancer of various organs (Essa et al. 2013; Heng et al. 2016; Badawy 2017; Lovelace et al. 2017; Sordillo et al. 2017; Boros et al. 2018; Platten et al. 2019).

The first step of the Kyn pathway can be catabolized by three distinct but functionally redundant enzymes: indoleamine 2,3-dioxygenase 1 (IDO1), IDO2, and tryptophan 2,3-dioxygenase (TDO2) (Metz et al. 2007; Löb et al. 2009). This step produces the intermediate N-formyl kynurenine, which is converted into Kyn by arylformamidase (AFMID). Kyn can be further metabolized to give rise to kynurenic acid, cinnabarinic acid, xanthurenic acid, picolinic acid, quinolinic acid, and NAD^+^ (Essa et al. 2013; Zhai et al. 2015b; Palego et al. 2016). Previous studies have found one or more of these enzymes were up-regulated in tumors of the pancreas, breast, and brain (Opitz et al. 2011; Adams et al. 2012, 2014; Taguchi et al. 2014; Liu et al. 2015; Heng et al. 2016). In colorectal cancer, IDO1 expression correlates with reduced tumor infiltration by lymphocytes, increased rates of hepatic metastases, and a poor clinical outcome (Brandacher et al. 2006). Recently, it was shown that conditional deletion of IDO1 in colonic cells delays colon cancer in animal models (Bishnupuri et al. 2019), indicating that Kyn levels are likely altered in colon cancer and may contribute to tumorigenesis.

Previous studies have focused on the role of Kyn as a tumor-secreted metabolite that inhibits cancer immune surveillance. Tumor cell-produced Kyn can be exported to the tumor microenvironment, leading to T-cell inactivation and prevention of tumor cell clearance (Opitz et al. 2011; Hughes et al. 2014; Zhai et al. 2015a,b; Liu et al. 2018). Kyn can also function as an endogenous ligand for the basic helix–loop–helix (bHLH)-PAS transcription factor aryl hydrocarbon receptor (AHR) (Opitz et al. 2011), suggesting a cell-autonomous role. Activation of AHR by tumor-produced Kyn is believed to elicit a gene expression program that not only results in the paracrine suppression of immune cells (Opitz et al. 2011) but also facilitates cancer cell proliferation and migration in a cell-autonomous manner (Bersten et al. 2013; Murray et al. 2014).

AHR expression is correlated with smoke-induced lung cancer and is believed to contribute to lung cancer initiation (Tsay et al. 2013). AHR is highly expressed in tumors of different origins, including lung, breast, liver, ovarian, prostate, and colon (Koliopanos et al. 2002; Opitz et al. 2011). Overexpression of a constitutively active AHR causes stomach cancer (Andersson et al. 2002). Moreover, studies of human colon cancer cells show that AHR activation by the dioxin TCDD drives colon cancer cell survival and migration (Xie et al. 2012). Recently, our laboratory discovered that the proto-oncogene MYC induces AHR expression in colon cancer (Lafita-Navarro et al. 2018). MYC is one of the most commonly overexpressed genes in human tumors and is required for maintenance of most cancers, including colon cancer (Brodeur et al. 1984; Nau et al. 1985; Sansom et al. 2007; Beroukhim et al. 2010). Pioneering studies found that MYC regulates genes that enhance amino acid uptake (Yue et al. 2017), including the uptake of glutamine, which, together with glucose, fuels the TCA cycle in proliferative cells (Yuneva et al. 2007; Wise et al. 2008; Hsieh et al. 2015).

AHR is highly expressed in human colon cancer tissues, where it is localized in the nuclei of tumor cells (Lafita-Navarro et al. 2018). Given the requirement for ligand binding to AHR for its translocation to the nucleus, this nuclear localization of AHR likely reflects an increase in intracellular ligands of AHR in cancer cells. Here, we show that MYC promoted an increase in Kyn by inducing the transcription of the Trp importers and the enzyme AFMID. Furthermore, we demonstrate that Kyn levels were elevated in colon cancer tissues and human colon cancer cells and that colon cancer cells were selectively sensitive to inhibition of the Kyn pathway. We propose that reducing the conversion of Trp into Kyn could be an effective strategy to limit the proliferation of MYC-dependent cancer cells.

## Results

### MYC expression promotes an increase in Kyn levels

**Figure 1.**
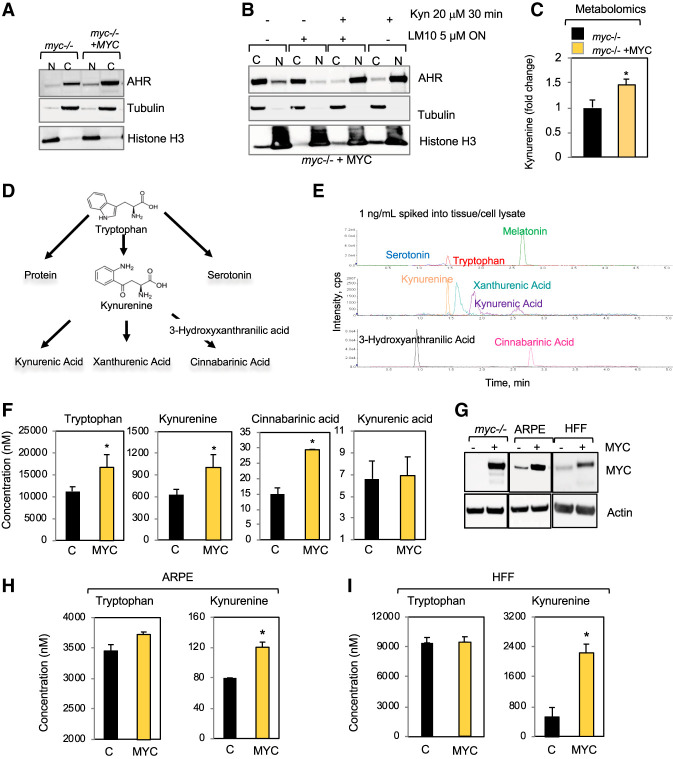
MYC induces the expression of Trp importers and AFMID. (*A*) Western blots for AHR comparing nuclear and cytoplasmic fractions of *myc*^−/−^ cells and *myc*^−/−^ cells reconstituted with MYC. (N) Nuclear fraction; (C) cytoplasmic fraction. (*B*) Nuclear and cytoplasmic fractions of *myc*^−/−^ cells expressing MYC and incubated with either DMSO or the TDO inhibitor LM10 at 5 µM overnight. DMSO or 20 µM Kyn was added 30 min prior to harvesting and fractionation. (*C*) Global metabolomics analyses compared *myc*^−/−^ cells expressing empty vector or reconstituted with MYC, and Kyn levels were plotted as fold change of values obtained in MYC-expressing cells over *myc*^−/−^ cells. (*D*) Schematic of the Trp metabolism pathway showing how Trp can be incorporated into protein or metabolized through the Kyn or serotonin pathways. (*E*) Method developed to measure the indicated Trp metabolites by high-performance liquid chromatography (HPLC)-tandem mass spectrometry (LC-MS/MS); the indicated Trp metabolites (1 ng/mL) were spiked into tissue/cell lysates, and the elution time for each was obtained. (*F*) Mass spectrometric measurements of Trp, Kyn, kynurenic acid, and cinnabarinic acid in *myc*^−/−^ cells expressing empty vectors or reconstituted with MYC. (*G*) MYC expression in *myc*^−/−^ cells, ARPE cells, and human foreskin fibroblasts (HFFs) expressing empty vector or MYC. (*H*,*I*) ARPE cells (*H*) and HFFs (*I*) were grown for 3 d, and then metabolites were extracted with methanol and subjected to LC-MS/MS to quantify the levels of Trp and Kyn. (*) *P* < 0.05.

As we showed previously, MYC expression increased the nuclear levels of AHR in multiple cells lines (Fig. 1A; Lafita-Navarro et al. 2018), thus indicating that AHR is transcriptionally active in these cells. We found that AHR target genes were indeed up-regulated in MYC-expressing fibroblasts (Lafita-Navarro et al. 2018). Incubation of these fibroblasts with Kyn and Trp induced nuclear translocation of AHR to an extent similar to that of TCDD and Indirubin, which are exogenous ligands for AHR (Supplemental Fig. S1A). Treating *myc*^−/−^ cells expressing MYC with the TDO/IDO inhibitor LM10 (Fig. 1B) or 680C91 (Supplemental Fig. S1B) limited nuclear translocation of AHR, which was rescued by the addition of Kyn to the culture medium. These results suggest that MYC-expressing cells have an increase in nuclear translocation of AHR due at least in part to increased levels of endogenous Kyn.

To determine whether MYC promotes an increase in the intracellular levels of Kyn, we applied metabolomics profiling to compare the global metabolites present in *myc*^−/−^ cells expressing empty vector or reconstituted with MYC. This experiment revealed that MYC expression promoted an increase in Kyn levels (Fig. 1C). To accurately quantify the levels of Trp metabolites in cells and tissues, we first developed a high-performance liquid chromatography (HPLC)-tandem mass spectrometry (LC-MS/MS) method using commercially available purified compounds (Fig. 1D) to generate individual standard curves for each metabolite (Fig. 1E). Using this method, we found that Trp, Kyn, and cinnabarinic acid were elevated upon MYC expression in *myc*^−/−^ cells (Fig. 1F). Kynurenic acid was unaffected by MYC expression, and xanthurenic acid and melatonin were undetectable. Trp levels were 20 times higher than Kyn, thus suggesting that a large portion of cellular Trp is available for protein synthesis. Cinnabarinic acid and kynurenic acid were present in much smaller amounts than Kyn (Fig. 1F). Serotonin levels were either reduced or enhanced by MYC, depending on the cell line (Supplemental Fig. S1C,D); thus, we concluded that MYC does not have a consistent effect on serotonin levels. Importantly, serotonin levels were lower than Kyn in epithelial cells and fibroblasts, as expected, since serotonin is produced by specialized cells (Supplemental Fig. S1C,D).

We extended our studies to compare Kyn levels in the epithelial human retinal epithelial cell line ARPE and normal human foreskin fibroblasts (HFFs) expressing empty vector or MYC (Fig. 1G). We found that Kyn levels were consistently elevated in both ARPE (1.5×) and HFF (5×) cells upon MYC expression as detected by LC-MS/MS (Fig. 1H,I). In summary, using independent approaches, we demonstrated that MYC-expressing cells displayed increases in intracellular Trp and Kyn. Kyn was the most abundant Trp metabolite in MYC-expressing cells, thus indicating that Kyn is a stable Trp catabolite and may play roles in one or more aspects of cellular behavior.

### MYC promotes the expression of Trp transporters and enzymes in the Kyn pathway

Oncogenic transformation by MYC (and other oncogenes) often leads to an increase in the uptake of essential amino acids (Wise et al. 2008; Yue et al. 2017). Therefore, increased Trp uptake is likely to occur in MYC-expressing cells to supply Trp for enhanced protein synthesis and for the Kyn pathway. Solute carrier 7A5 (SLC7A5; also named LAT1) and SLC1A5 were shown previously to import Trp (Patel et al. 2013; Ylikangas et al. 2014; Cibrian et al. 2016; Timosenko et al. 2016; Cormerais et al. 2018). SLC7A5 has high affinity for large neutral amino acids such as phenylalanine, tyrosine, leucine, arginine, and Trp when associated with SLC3A2 (Scalise et al. 2018). SLC1A5 is known as a glutamine transporter but can also import alanine, threonine, serine, leucine, valine, asparagine, methionine, isoleucine, Trp, histidine, and phenylalanine but not glutamate, lysine, arginine, and branched chain amino acids (Pochini et al. 2014).

**Figure 2.**
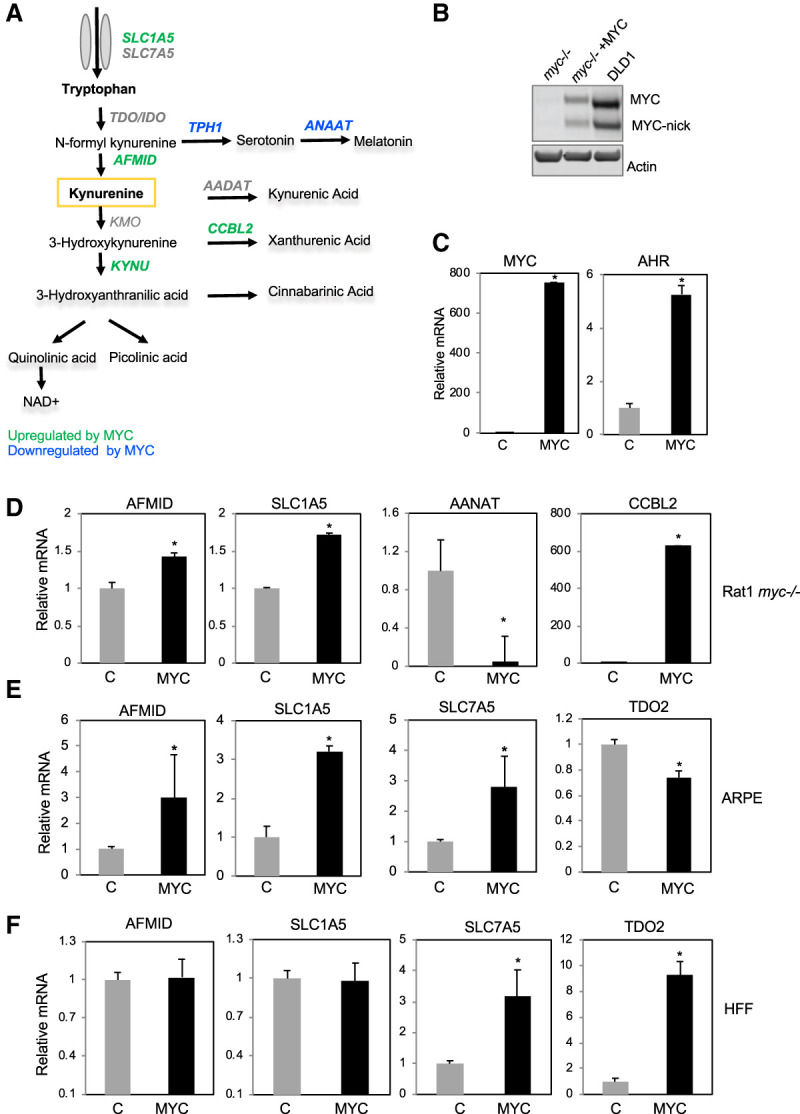
MYC regulates the expression of Trp transporters and enzymes in the Kyn pathway. (*A*) Trp pathway displaying genes up-regulated by MYC in green and genes down-regulated by MYC in blue as identified by RNA-seq comparing *myc*^−/−^ cells expressing empty vector or MYC. (*B*) Western blot for MYC in lysates of *myc*^−/−^ cells expressing empty vector or MYC and the DLD1 colon cancer cell line. (*C*) MYC and AHR mRNA expression in *myc*^−/−^ cells expressing empty vector or MYC. (*D*) RT-qPCR comparing the expression of the indicated genes in the Kyn pathway and in the serotonin pathway in *myc*^−/−^ cells expressing empty vector or MYC. (*E*) RT-qPCR for the expression of the indicated Trp transporters and enzymes in the Kyn pathway in ARPE cells expressing empty vector or MYC. (*F*) RT-qPCR for the expression of the indicated Trp transporters and enzymes in the Kyn pathway in HFF cells expressing empty vector or MYC. (*) *P* < 0.05.

To interrogate the mechanism by which MYC promotes Trp and Kyn accumulation, we probed RNA sequencing (RNA-seq) data comparing the profile of *myc*^−/−^ cells expressing empty vector or reconstituted with MYC, which we published previously (Lafita-Navarro et al. 2018). A comparison of data from two independent RNA-seq experiments in *myc*^−/−^ cells expressing empty vector or MYC revealed MYC-regulated genes related to Trp uptake and metabolism (Fig. 2A; Supplemental Fig. S1E). MYC induced the expression of SLC1A5 and the Kyn pathway enzymes AFMID, KYNU, and CCBL2 and repressed the expression of the enzymes TPH1 and AANAT (Fig. 2A). SLC7A5, shown previously to be a target of MYC, was not induced in our RNA-seq data. The modulation of these genes by MYC was validated by performing RT-qPCR in *myc*^−/−^ cells expressing empty vector or reconstituted with MYC (Fig. 2B). MYC and AHR (a MYC target gene) (Lafita-Navarro et al. 2018) were used as controls in our RT-qPCR experiments (Fig. 2C).

We examined the expression of Trp transporters and Trp-metabolizing enzymes in HFF and ARPE cells upon MYC expression. Using RT-qPCR, we found that AFMID and SLC1A5 were also induced by MYC in ARPE (Fig. 2E), similarly to *myc*^−/−^ cells (Fig. 2D), but not in HFFs expressing MYC (Fig. 2F). To determine whether MYC expression regulated the expression of other Trp transporters or enzymes in the Kyn pathway in HFFs, we examined the expression of the enzymes IDO1 and TDO2 in HFFs. We found that TDO2 was elevated in HFFs expressing MYC but not in ARPE (Fig. 2E,F). SLC7A5, which is not controlled by MYC in Rat1 *myc*^−/−^ fibroblasts, is a known MYC target gene (Yue et al. 2017) and was elevated upon MYC expression in ARPE (Fig. 2E) and HFF (Fig. 2F) cells. Interestingly, each cell line displayed an increase in at least one Trp importer and one enzyme in the Kyn pathway upon MYC overexpression, thus indicating that MYC promotes the expression of a combination of transporters and enzymes necessary to increase uptake of Trp and its conversion into Kyn. The genes that could not be validated by RT-qPCR as a result of very low mRNA expression and thus prevented reliable conclusions about their relative expression are not shown.

### Colon cancer tissues have elevated Trp transporters and enzymes in the Kyn pathway

**Figure 3.**
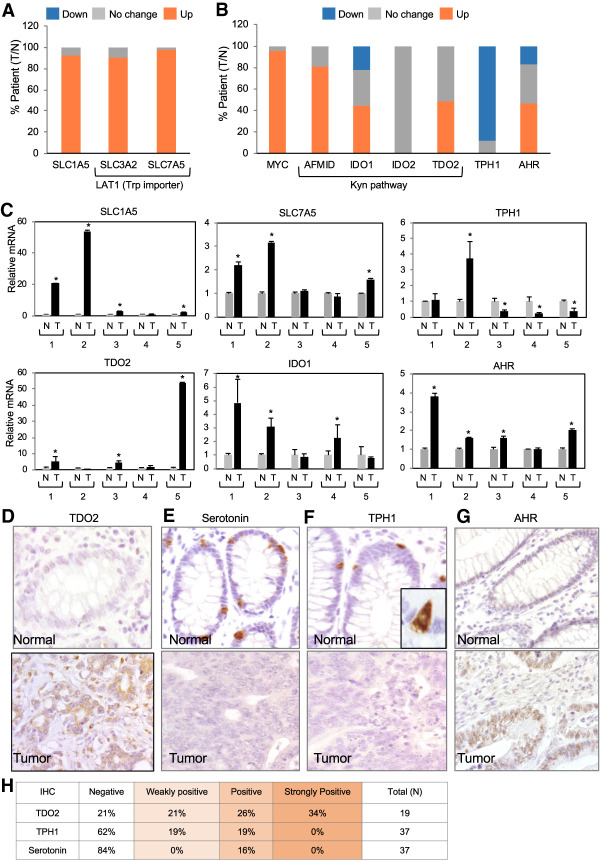
L-amino acid transporters that import Trp and enzymes in the Kyn pathway are elevated in colon cancer. (*A*,*B*) Fold change in mRNA levels of Trp transporters and enzymes involved in the Trp pathway in human colon adenocarcinomas obtained from the TCGA database. Comparisons are between normal and tumor tissues from the same patient. Down-regulation indicates a <0.7-fold change, no change indicates a 0.7-fold to 1.3-fold change, and up-regulation indicates a >1.3-fold change. (*C*) RT-qPCR for the expression of the indicated Trp transporters and enzymes in the Kyn pathway in human normal tissues and colon adenocarcinomas. Gene expression was normalized to the total cDNA. (*D*) IHC for TDO in a representative human colon cancer patient sample and adjacent normal tissue. (*E*) IHC for serotonin in a representative human colon cancer patient sample and adjacent normal tissue. (*F*) IHC for TPH1 in a representative human colon cancer patient sample and adjacent normal tissue. (*G*) IHC for AHR in a representative human colon cancer patient sample and adjacent normal tissue. (*H*) Quantification of IHC for TDO2, TPH1, and serotonin. (*) *P* < 0.05.

By analyzing the mRNA expression of SLC1A5, SLC3A2, and SLC7A5 in human colon cancer samples deposited in The Cancer Genome Atlas (TCGA) database (*n* = 41 matched pairs of normal and colorectal cancer samples) (Supplemental Table S1), we found that all three genes had elevated expression in nearly all patients (Fig. 3A). Indeed, previous immunohistochemistry (IHC) studies found that both SLC1A5 and SLC7A5 were up-regulated in colon cancer cells (Huang et al. 2014; Wang et al. 2016; Toda et al. 2017).

We also probed normal and tumor tissues in the TCGA database for the expression of Trp-metabolizing enzymes. We found that the enzymes IDO1 and TDO2 were elevated in ∼40% of the samples from colon cancer patients, and the enzyme AFMID, which is involved in the last step of the conversion of Trp into Kyn, was up-regulated in 80% of these samples of colon cancers (Fig. 3B). The enzyme TPH1, which is involved in the production of serotonin, was down-regulated in ∼90% of the patient samples (Fig. 3B). To validate these results, we performed RT-qPCR for SLC1A5, SLC7A5, TPH1, TDO2, IDO1, AHR (Fig. 3C), and MYC (Supplemental Fig. S2A) in colon cancer and normal tissue of the same patients. Our results confirmed that SLC7A5, SLC1A5, TDO2, IDO1, and AHR were all elevated in colon cancer, while TPH1 was reduced (Fig. 3C).

We performed IHC for TDO2, TPH1, AHR, serotonin, and TPH2 in paraffin-embedded patient-derived normal and colon cancer tissues to confirm our TCGA results. Antibodies for AFMID and IDO1/2 did not yield specific signals in human colonic tissues. All other samples were characterized into four groups: negative, weakly positive, positive, and strongly positive (example in Supplemental Fig. S2H). TDO2 expression was significantly higher in 15 out of 18 samples (Fig. 3D,H; Supplemental Fig. S2D). Importantly, when comparing nuclear AHR and TDO expression, most patient samples had elevated TDO2 and nuclear AHR (Supplemental Fig. S2G), thus indicating a correlation between nuclear translocation of AHR and Kyn synthesis. Most patients had little to no TPH1 and its product serotonin in their tumor samples, while nearby normal tissue displayed TPH1-positive cells (Fig. 3E,F), which are secretory epithelial cells specialized in producing serotonin (Bornstein 2012; Gershon 2012; Baganz and Blakely 2013), named enterochromaffin cells (ECs) (Supplemental Fig. S2C,E). TPH2, which is normally expressed in enteric neurons, was indeed absent in both normal and tumor tissue (Supplemental Fig. S2F). AHR expression was also elevated in colon cancer samples (Fig. 3G), as reported previously by our laboratory (Lafita-Navarro et al. 2018). Elevated levels of the enzymes TDO2, IDO1, and AFMID combined with the Trp transporters SLC1A5 and SLC7A5 in colon cancer patients may lead to increased Trp and Kyn levels in colon cancer cells.

### Colon cancer cell lines display increased Trp importers and enzymes in the Kyn pathway

**Figure 4.**
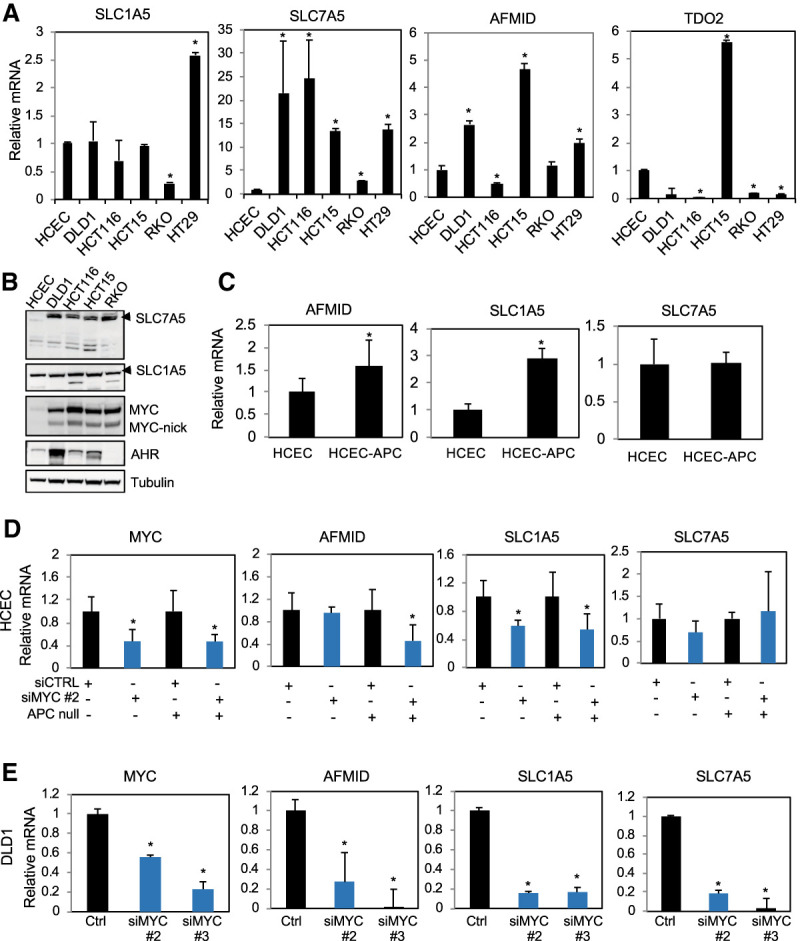
Colon cancer cell lines have elevated levels of Trp importers, Trp-metabolizing enzymes, and Kyn. (*A*) RT-qPCR for the expression of the indicated genes and cell lines. (*B*) Western blot for SLC7A5, SLC1A5, MYC, and AHR in total lysates of colon cancer cell lines and normal HCECs grown for 3 d. (*C*) RT-qPCR for the indicated genes in HCECs and HCECs expressing a loss-of-function mutation on APC, which leads to activation of the WNT pathway. (*D*) RT-qPCR of the indicated genes in HCECs and HCEC-APCs transfected with control siRNA or siRNA targeting MYC. Fold change was determined by normalizing mRNA levels obtained from cell transfected with siRNA for MYC by mRNA levels of cells transfected with control siRNA. (*E*) RT-qPCR of the indicated genes in DLD1 cells transfected with control siRNA or with siRNA targeting MYC. Fold change was obtained as described in *D*.

To determine whether the Trp importers, Trp-metabolizing enzymes, and Trp catabolites were up-regulated in colon cancer cells, we compared their levels in DLD1, HCT116, HCT15, RKO, and HT29 colon cancer cells lines with those in h-Tert immortalized normal human colonic epithelial cells (HCECs). In agreement with the data obtained from human samples in the TCGA database, all colon cancer cell lines had elevated levels of the SLC7A5 transporter (Fig. 4A). Surprisingly, only one colon cancer cell line displayed an increase in SLC1A5 when compared with HCECs (Fig. 4A). The Trp-metabolizing enzyme AFMID was elevated in three and reduced in one of the five colon cancer cell lines examined (Fig. 4A). TDO2 was reduced in all of the cancer cells lines except HCT15 (Fig. 4A). IDO1 mRNA expression was extremely low in all lines, preventing reliable conclusions on its relative levels (data not shown). Overall, human colon cancer samples displayed a more robust up-regulation in the expression of Trp transporters and enzymes in the Kyn pathway than cultured colon cancer cell lines, potentially indicating that this pathway is more active in vivo. Nevertheless, cultured colon cancer cell lines examined here had an increase in SLC7A5 and AFMID, indicating that the Kyn pathway is active.

SLC7A5 was the most consistently up-regulated gene in the Trp pathway in cultured colon cancer cell lines, and Western blots confirmed that SLC7A5 protein was dramatically up-regulated in colon cancer cells, while changes in SLC1A5 were more modest (Fig. 4B). To determine whether Trp-related genes are induced during the initial steps of cellular transformation, we compared HCECs and HCECs transformed by the expression of a mutant form of APC that leads to its loss of its tumor suppressive function: the activation of the WNT pathway. Activation of the WNT pathway drives colon cancer in mice in a MYC-dependent manner (Sansom et al. 2007). We found that the MYC target genes AFMID and SLC1A5, but not SLC7A5, were elevated upon transformation by mutant APC in HCECs (Fig. 4C). To determine whether MYC is responsible for the regulation of these genes, we used siRNA to knock down MYC in HCECs and HCEC-APCs and found that AFMID and SLC1A5 were down-regulated (Fig. 4D). To determine whether MYC regulated the expression of Trp-related genes in established colon cancer cells, we knocked down MYC in DLD1 cells. We found that MYC knockdown dramatically reduced the expression of SLC1A5, SLC7A5, and AFMID (Fig. 4E). Taken together, our results indicate that Trp transporters and Trp-metabolizing enzymes are up-regulated by MYC in colon cancer cells.

### MYC increases Trp uptake and metabolism in colonic cells

**Figure 5.**
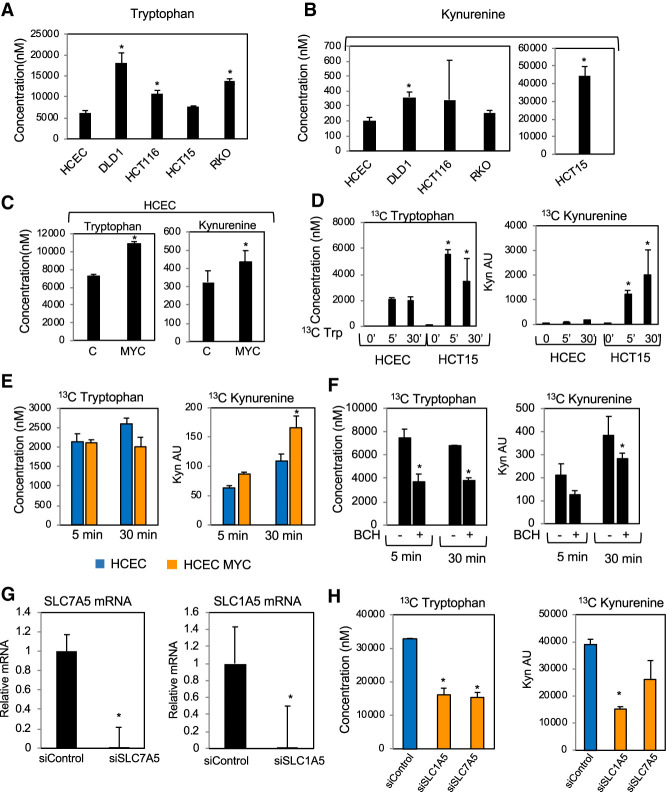
Trp metabolites in the Kyn pathway are elevated in colon cancer cell lines. (*A*) LC-MS/MS quantification of Trp levels in colon cancer cell lines and HCECs. Cells were harvested 72 h after plating, and metabolites were extracted with 80% methanol. The pellet was used to measure total protein concentration, and the amounts of metabolites were normalized to protein levels from the same sample. (*B*) LC-MS/MS quantification of Kyn levels in colon cancer cell lines and HCECs grown as in *A*. (*C*) LC-MS/MS quantification of Trp and Kyn in HCECs and HCECs expressing MYC. (*D*) LC-MS/MS quantification of cellular levels of ^13^C-Trp (^13^C_11_-L-tryptophan) and ^13^C-Kyn in HCECs and HCT15 cells measured after 5 and 30 min of incubation. A total of 1 × 10^6^ cells was plated in DMEM containing 1% serum, and, 24 h later, the medium was replaced by Trp-free DMEM containing 1% serum, which was supplemented with 75 µM ^13^C-Trp. Metabolites were extracted with 80% methanol, and the pellet was used to quantify total protein in each sample. (*E*) LC-MS/MS quantification of ^13^C-Trp and ^13^C-Kyn in HCECs and HCECs expressing MYC 5 and 30 min after the addition of ^13^C-Trp. (*F*) LC-MS/MS quantification of the cellular levels of ^13^C-Trp and ^13^C-Kyn in HCT15 cells. Cells treated as in *D* were incubated with the L-amino acid transporter inhibitor BCH or DMSO for 5 or 30 min, harvested, and used to quantify ^13^C-Trp and ^13^C-Kyn. (*G*) RT-qPCR for SLC1A5 and SLC7A5 in HCT15 cells transfected with control siRNA or siRNA targeting SLC1A5 and SLC7A5. (*H*) LC-MS/MS quantification of cellular levels of ^13^C-Trp and ^13^C-Kyn in HCT15 cells transfected with either control siRNA or siRNA targeting SLC1A5 and SLC7A5. Cells were transfected with siRNA and, 72 h later, incubated with ^13^C-Trp for 1 h, and the levels of ^13^C-Trp and ^13^C-Kyn were measured.

Comparing colon cancer cell lines and HCECs, we demonstrated that all colon cancer cell lines had elevated Trp (Fig. 5A). This agrees with the elevated levels of Trp transporters in colon cancer cell lines and tissues. Kyn was also elevated in colon cancer cell lines (Fig. 5B). Cinnabarinic acid was not significantly altered in colon cancer cell lines apart from HCT15 and HT29, which had reduced levels (Supplemental Fig. S3A). Xanthurenic acid was elevated in HT29 and DLD1 cells, while kynurenic acid levels were undetectable (Supplemental Fig. S3B). Interestingly, HCT15 displayed the highest levels of AFMID and Kyn but lowest Trp (Figs. 4A, 5A,B), thus suggesting that the Kyn pathway is very active in cell lines with characteristics of HCT15. To determine whether MYC is responsible for increased Trp and Kyn in colon cancer cells, we expressed MYC in HCECs and quantified Trp and Kyn by LC-MS/MS and found that MYC expression caused an increase in Trp and Kyn in HCECs (Fig. 5C).

To better understand Trp metabolism in tumors, we measured the incorporation and processing of stable nonradioactive ^13^C-labeled Trp in colon cancer cells (Supplemental Fig. S3C). First, we compared the ability of HCECs and HCT15 (which express elevated levels of Trp importers and Trp-metabolizing enzymes) to take up and metabolize Trp by the Kyn pathway. We incubated these cells with Trp isotopically labeled in all carbons (^13^C-Trp [^13^C_11_-L-tryptophan]) for 5 or 30 min (Supplemental Fig. S3D). Methanol-extracted metabolites underwent LC-MS/MS measurement to quantify ^13^C-Trp and ^13^C-Kyn (Supplemental Fig. S4E). ^13^C-Trp was present at a higher concentration in the HCT15 cell line within 5 min after the addition of ^13^C-Trp to the culture medium (Fig. 5D). This agrees with the higher levels of Trp importers in HCT15 cells than in HCECs (Fig. 4A). ^13^C-Kyn levels were also elevated after a 5-min incubation with ^13^C-Trp (Fig. 5D), thus indicating that the conversion of Trp into Kyn is a rapid process. To confirm that transformation of colonic cells by MYC is sufficient to alter the conversion rates of Trp into Kyn, we compared Trp metabolism in HCECs expressing empty vector or MYC. We found that overexpression of MYC increased the conversion of ^13^C-Trp into ^13^C-Kyn (Fig. 5E), thus demonstrating that MYC expression regulates Kyn levels in colonic cells.

To determine whether Trp uptake affects the production of Kyn, we incubated HCT15 cells with ^13^C-Trp for 5 and 30 min in the presence of DMSO or BCH, an inhibitor of L-amino acid import. BCH limited the Trp uptake and reduced the levels of ^13^C-Trp and ^13^C-Kyn but did not prevent the conversion of Trp into Kyn (Fig. 5F). Silencing SLC1A5 or SLC7A5 in the colon cancer cell line HCT15 (Fig. 5G) led to a reduction of ^13^C-Trp and ^13^C-Kyn observed 1 h after addition of ^13^C-Trp to the culture medium (Fig. 5H). Because SLC1A5 and SLC7A5 transport several amino acids other than Trp, we asked whether other amino acids compete with Trp for import from the culture medium and whether the presence of other amino acids affects Kyn production. We measured the levels of cellular Trp in HCT15 cells incubated with minimum essential amino acids (MEM) or MEM in combination with nonessential amino acids (NEAAs) and found no difference in the levels of Trp in these two conditions (Supplemental Fig. S4B). We also measured the uptake of ^13^C-Trp in cells grown in amino acid-free medium alone or in the presence of NEAAs or glutamine, which is transported by SLC1A5 (Supplemental Fig. S4B). We found that providing glutamine does not affect the uptake of ^13^C-Trp, thus indicating that Trp does not compete with glutamine for amino acid transporter availability (Supplemental Fig. S4B). Adding a mixture of NEAAs did cause a modest reduction in Kyn (Supplemental Fig. S4A) and Trp, which was not statistically significant. Our results indicate that Trp uptake was not significantly affected by the presence of other amino acids, thus suggesting that, under the culture conditions used in our experiments, Trp does not compete with other amino acids to enter cancer cells. However, it is possible that in vivo amino acid competition does play a role in the selective uptake of Trp.

To probe the importance of AFMID to the production of Kyn in colon cancer cells, we knocked down AFMID in colon cancer cells and measured Kyn. Surprisingly, we found that AFMID knockdown led to a reproducible but marginal reduction in Kyn synthesis that did not always meet statistical significance (Supplemental Fig. S4C,D). Given that AFMID is necessary for Kyn synthesis (Fig. 2A), it is possible that siRNA-mediated knockdown was not sufficient to significantly impair AFMID activity. Attempts to generate colon cancer cells lines that have AFMID knocked out have not been successful. Moreover, previous studies based on AFMID knockout mice have suggested that other, yet uncharacterized genes may compensate for the loss of AFMID in some organs, such as the kidneys (Dobrovolsky et al. 2005). Further studies targeted at eliminating AFMID function and investigating the cross-talk between AFMID and other metabolic enzymes will better delineate the role of this enzyme in cancer cells. Taken together, our results suggest that increased Kyn in MYC-dependent cells is caused by augmented Trp uptake and possibly by AFMID-mediated conversion of Trp into Kyn.

### Colon cancer tissues have elevated Kyn

**Figure 6.**
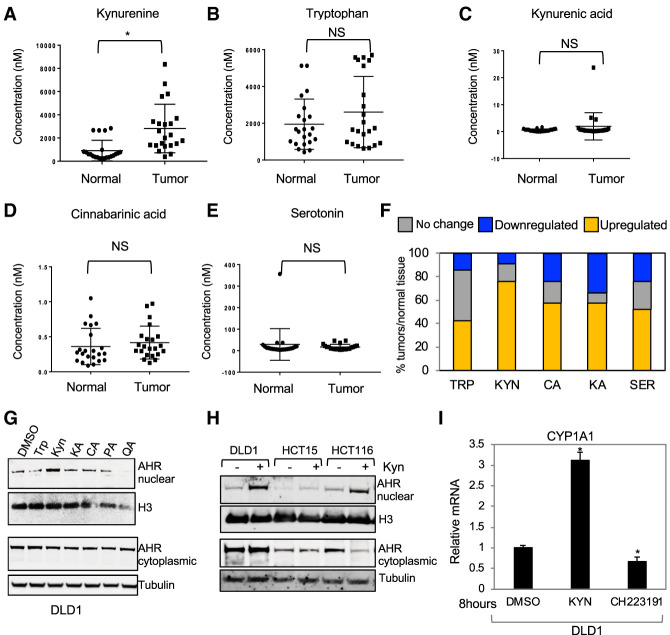
Trp metabolites in the Kyn pathway are elevated in human colon cancer tissues. (*A*–*E*) LC-MS/MS measurements of levels of Kyn, Trp, kynurenic acid, cinnabarinic acid, and serotonin in normal human colon and tumor adenocarcinomas. (*F*) Fold change in levels of metabolites measured in *A*–*E*. Comparisons are between normal and tumor tissues from the same patient. Down-regulation indicates a <0.7-fold change, no change indicates a 0.7-fold to 1.3-fold change, and up-regulation indicates a >1.3-fold change. (*) *P* < 0.05. (*G*) Western blotting of the nuclear and cytoplasmic fractionations of DLD1 cells treated with 20 µM Trp metabolites for 20 min. (KA) Kyn acid; (CA) cinnabarinic acid; (PA) picolinic acid; (QA) quinolinic acid. (*H*) Western blotting for nuclear and cytoplasmic fractions of the indicated cells incubated with 20 µM Kyn for 20 min. (*I*) RT-qPCR for CYP1A1 in DLD1 cells incubated with DMSO, 20 µM Kyn, or 10 µM CH223191. Fold change was obtained by comparing mRNA levels obtained in treated samples by DMSO control.

To determine whether Trp metabolism is altered in colon cancer, we measured the levels of Trp and its metabolites in human colon cancer and normal tissues using the method described in Figure 1, D and E. We found that colon cancer tissues had Kyn levels that were ≥1.5-fold higher than their surrounding normal mucosa, while Trp levels were more variable (Fig. 6A,B). Using the same LC-MS/MS platform, we also measured the levels of other stable Trp metabolites downstream from Kyn (kynurenic acid, cinnabarinic acid, xanthurenic acid, and 3-hydroxyantharanilic acid) in addition to serotonin and melatonin. Colonic tissue had undetectable levels of melatonin and 3-hydroxyxantharanilic acid (data not shown), while kynurenic acid levels were 100 times lower than Kyn (Fig. 6C), and cinnabarinic acid was >1000 times lower than Kyn (Fig. 6D). Serotonin levels were ∼20 times lower than Kyn (Fig. 6E). Levels of Kyn were variable among patients; however, when normalizing Kyn concentrations in tumors by normal mucosa of the same patients, Kyn was elevated in nearly 80% of the patients (Fig. 6F). Cinnabarinic acid, kynurenic acid, and serotonin levels were elevated in ∼50% of the patients (Fig. 6F). Kyn was present at much a higher concentration than other Trp metabolites in colon cancer, and Trp and Kyn were found at equimolar concentrations in colon cancer (Supplemental Fig. S4E). Our method provides a quantitative tool to determine absolute levels of Trp metabolites in human tissues, and our results agree with a previous study showing that colon cancer patients display an increase in circulating levels of Trp metabolites and increased IHC signal with an anti-Kyn antibody (Puccetti et al. 2015).

The best-studied role for Kyn is its ability to function as a ligand of AHR (DiNatale et al. 2010; Opitz et al. 2011; Adams et al. 2012; Badawy 2017). Therefore, we asked whether Kyn regulates AHR activity in colon cancer cells. Because Kyn is a precursor of downstream catabolites, we compared Kyn, kynurenic acid, cinnabarinic acid, picolinic acid, and quinolinic acid for their ability to increase the nuclear pool of AHR after 20 min of incubation. We found that only Kyn was capable of inducing nuclear translocation of AHR in colon cancer cells (Fig. 6G). These data agree with data from a study showing that Kyn is the only Trp metabolite capable of inducing activation of an AHR reporter plasmid, as shown previously in other cell types (Mezrich et al. 2010). Treating additional colon cancer cells with Kyn also promoted AHR nuclear translocation (Fig. 6H) and induced the expression of the canonical of AHR target gene CYP1A1 (Fig. 6I). These results indicated that Kyn is elevated in colon cancer, where it activates signaling via AHR.

### Colon cancer cells are sensitive to inhibitors of the Kyn pathway

**Figure 7.**
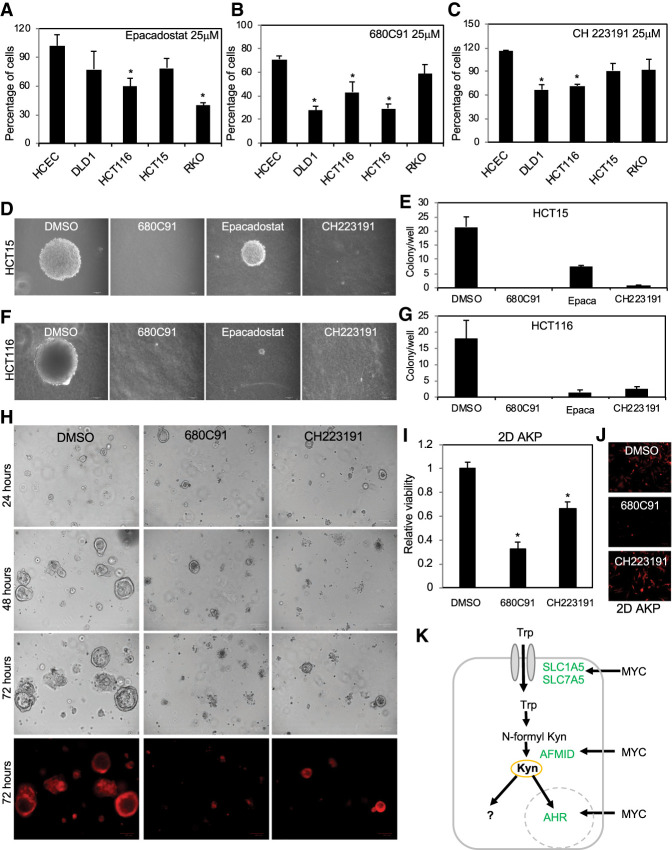
Blocking the Kyn pathway leads to preferential death of colon cancer cells. (*A*) Cells were treated with 25 µM epacadostat for 3 d in DMEM containing 1% serum. Cell viability was calculated in relationship to DMSO-treated controls from the same cell line. (*B*) Cells were treated with 25 µM 680C91 for 3 d in DMEM containing 1% serum. Cell viability was calculated in relationship to DMSO-treated controls from the same cell line. (*C*) Cells were incubated with 25 µM CH223191 for 3 d in DMEM containing 1% serum, and cell viability was determined in relationship to DMSO-treated controls from the same cell line. (*D*) Bright-field images of HCT15 cells grown in soft agar and incubated with DMSO or 25 µM 680C91, epacadostat, or CH223191 for 4 wk in DMEM containing 1% serum. (*E*) Quantification of the colonies from *D* was performed by staining the samples with crystal violet and counting all colonies regardless of their size. (*F*) Bright-field images of HCT116 cells grown as in *D*. (*G*) Quantification of the colonies in *F*. (*H*) APC^−/−^ (CRISPR), p53^−/−^ (floxed allele, excised), KRas^G12D^ (activated from LSL-KRas^G12D^), and TdTomato red mouse organoids (AKP) grown in Matrigel were incubated with DMSO, 25 µM 680C91, or 10 µM CH223191. Bright-field images of representative colonies grown in Matrigel at 24, 48, and 72 h after addition of drugs. Red fluorescent channel shows the expression of TdTomato red reporter. (*I*) The 2D AKP cell line was obtained by growing organoids attached to tissue culture plates and served as an isogenic culture for the 3D experiments. These cells were incubated with 25 µM 680C91 or 10 µM CH223191 for 2 d, and the relative viability was obtained by normalizing the cell numbers in the samples treated with either 680C91 or CH223191 by DMSO controls. (*J*) The red fluorescent channel shows the expression of TdTomato red reporter in 2D AKP cells. (*K*) Model for increased Trp uptake and processing in colon cancer cells. MYC increases the expression of the Trp transporters SLC1A5 and SLC7A5 and the enzyme AFMID. Trp taken up by cancer cells is used for de novo protein synthesis and to produce the metabolites in the Kyn pathway. Kyn functions as a ligand for the transcription factor AHR that translocates into the nucleus. (*) *P* < 0.05.

To determine the importance of Kyn to the growth of colon cancer cells, we compared the growth of normal HCECs and colon cancer cell lines in the presence of pharmacological inhibitors of TDO2 (680C91) and IDO (epacadostat). For these experiments, HCECs and colon cancer cell lines were seeded at low density, and, 24 h later, the culture medium was replaced by medium containing the indicated inhibitors. Cells were quantified 3 d later (Fig. 7A,B; Supplemental Figs. S5–S7). Colon cancer cells lines were more sensitive to TDO2 and IDO inhibition than HCECs (Fig. 7A,B). HCT15 cells expressed high levels of TDO2 (Fig. 4A) and were more sensitive to TDO2 inhibition than IDO inhibition (Fig. 7A,B). To determine whether this reduction in cell number was caused by cell death or cell cycle arrest, we performed cell cycle analysis using flow cytometry. We treated colon cancer cells lines with epacadostat or 680C91 for 3 d, after which the cells were harvested and stained with propidium iodide (PI) (Supplemental Fig. S5A,B). Treatment with inhibitors of IDO and TDO led to cell death and/or cell cycle arrest (Supplemental Fig. S5A,B). These inhibitors also reduced the levels of nuclear AHR in colon cancer cells, such as DLD1 (Supplemental Fig. S8A), as expected.

Because NAD^+^ is the final product of Kyn degradation, we asked whether NAD(H) levels were globally altered by treatment with TDO/IDO inhibitors and whether NAD(H) reconstitution was capable of rescuing cell death caused by these inhibitors. To determine the contribution of the Kyn pathway to NAD^+^ levels, we measured NAD(H) in HCT15 and DLD1 colon cancer cells treated with the inhibitors of TDO2 and IDO for 24 h (Supplemental Fig. S9A–C). H_2_O_2_, used as a control in our experiments, caused a dramatic reduction in NAD(H) levels, as expected (Supplemental Fig. S9A–C). However, incubation of DLD1 and HCT15 cells with 680C91 or epacadostat had no effect on the total NAD(H) levels, thus indicating that the Kyn pathway does not significantly contribute to NAD^+^ production in these colon cancer cell lines under the culture conditions used in our experiments. We also attempted to rescue cell death caused by 680C91, epacadostat, and the AHR inhibitor CH223191 with the NAD precursor nicotinamide mononucleotide (NMN). We found that NMN modestly increased the growth of some colon cancer cells incubated with DMSO or CH223191 but did not rescue cell death caused by the IDO/TDO inhibitors (Supplemental Fig. S9D–F). We interpreted these results to indicate that NMN facilitates the growth of some colon cancer cells (Supplemental Fig. S9) but cannot specifically prevent cell death caused by TDO and IDO inhibition.

To determine whether the growth-promoting ability of Kyn relies on AHR activation, we used the inhibitor of AHR, CH223191, which prevents the binding of AHR to its ligands (Zhao et al. 2010; Choi et al. 2012; Mohammadi-Bardbori et al. 2019). Treating colon cancer cells (such as DLD1) that contain AHR in the nucleus (Supplemental Fig. S8B) with CH223191 antagonized nuclear translocation of AHR by Kyn (Supplemental Fig. S8C). Indeed, CH223191 was sufficient to down-regulate the expression of the AHR target gene CYP1A1 (Fig. 6I). Importantly, colon cancer cells were more sensitive than HCECs to incubation with CH223191 (Fig. 7C). To determine whether this decrease in cell number was caused by cell death or reduced proliferation, we examined the cell cycle distribution by flow cytometry of cells treated with CH223191 and found that treatment with CH223191 led to cell cycle arrest (Supplemental Fig. S5).

We then tested the effects of epacadostat, 680C91, and CH223191 on the growth of the colon cancer cells HCT15 and HCT116 in soft agar. For this experiment, single cells were embedded in 0.3% agar and medium containing 1% serum with the inhibitors. Colonies were quantified 4 wk later and demonstrated that, as for 2D cultures, 3D soft agar-growing cells are sensitive to inhibition of the Kyn–AHR pathway (Fig. 7D–G). 3D cultures were more sensitive to CH223191 than cells grown in 2D. For example, HCT15 cells were not affected by CH223191 in 2D culture, but their growth was prevented by the treatment of these cells when grown in soft agar (Fig. 7D,E), indicating that the Kyn–AHR pathway may play a more prevalent role in cells grown in 3D culture and in vivo.

Organoids have become powerful tools to recapitulate critical aspects of tissue architecture and function in vitro (Date and Sato 2015; Mihaylova et al. 2018). Therefore, we used mouse colonic organoid cultures transformed by the loss of APC, knockout of P53, and expression of mutant KRAS (AKP) to determine the importance of the Kyn pathway. For this experiment, we plated equal cell numbers, allowed organoids to grow for 24 h, and then replaced the culture medium to contain either DMSO, epacadostat, 680C91, or CH223191. We found that all three drugs caused a decrease in organoid size and number (Fig. 7H). Similar results were obtained when growing these AKP organoids attached to tissue culture plates as 2D cultures (Fig. 7I,J), thus suggesting that our 2D results are informative and may reflect in vivo sensitivity to inhibition of the Kyn pathway. Importantly, as for the 3D soft agar growth, AKP cells grown as 3D cultures were more sensitive to CH223191 than cells in 2D.

Because colon cancer cells had an increase in Trp transporters (Fig. 3B) and elevated intracellular Trp and Kyn, we asked whether colon cancer cell lines have increased demand for Trp to support proliferation. We cultured colon cancer cell lines and HCECs in complete medium or medium lacking Trp for 3 d and found that colon cancer cell lines were, in general, more sensitive than HCECs to Trp depletion (Supplemental Fig. S10A). Flow cytometry cell cycle analyses of DLD1 colon cells grown in Trp-free medium for 3 d demonstrated an increased apoptosis (Supplemental Fig. S10B), thus confirming that Trp starvation promotes cell death in colon cancer cells. Silencing the Trp transporters SLC1A5 and SLC7A5 also reduced the viability of DLD1, HCT15, and HCT116 cells (Supplemental Fig. S10C). As expected, MYC-expressing cells, similar to transformed colon cancer cells, were more sensitive than *myc*^−/−^ cells to Trp depletion (Supplemental Fig. S10D). In addition, blocking TDO2 function with the inhibitor LM10 caused the preferential death of MYC-expressing cells (Supplemental Fig. S10E), thus suggesting that cells overexpressing MYC depend on Kyn. Thus, our results indicate that Kyn is necessary to sustain the proliferative state of colon cancer cells at least in part by promoting AHR nuclear translocation and transcriptional activation.

## Discussion

Our results show that colon cancer cells have an increased ability to obtain Trp and to process it by the Kyn pathway. This is driven by increased expression of Trp transporters (such as SLC7A5 and SLC1A5) and potentially the enzymes in the Kyn pathway (AFMID) (Fig. 7K). We demonstrated that both uptake and processing of Trp can be enhanced by the overexpression of a single oncogene: MYC. MYC expression is sufficient to induce the transcription of one or both Trp transporters and enzymes in the Kyn pathway. While the specific Trp transporters and enzymes induced by MYC varied according to cell types, we invariably observed that at least one transporter and one of the enzymes involved in converting Trp into Kyn were up-regulated by MYC expression in various cell types.

Aberrant up-regulation of any MYC family member (MYC, MYCN, or MYCL) promotes metabolic reprograming that leads to sustained proliferation of cancer cells (Brodeur et al. 1984; Nau et al. 1985; Hatton et al. 1996; Beroukhim et al. 2010; Liu et al. 2012; Hsieh et al. 2015). Because MYC is a universal oncogene, there have been several attempts to develop inhibitors that block MYC functions specifically in tumors (Soucek et al. 1998; Filippakopoulos et al. 2010; Hurlin 2013; Hart et al. 2014; McKeown and Bradner 2014; Abedin et al. 2016; Alghamdi et al. 2016; Zhu et al. 2017). However, MYC is still considered an undruggable protein (McKeown and Bradner 2014).

A novel and promising approach to target MYC-dependent cancer cells is based on interfering with metabolic pathways triggered by hyperactivated MYC. Seminal studies have shown that MYC regulates genes known to enhance glutamine and glucose uptake and processing to fuel the TCA cycle and to produce building blocks for proliferation (Yuneva et al. 2007; Wise et al. 2008). Indeed, glutamine was proposed to function as an essential amino acid specifically in cancer cells (Hsieh et al. 2015). While altered glutamine metabolism is well documented in tumors from multiple origins, the cancer-specific alterations in the metabolism of essential amino acids are not fully understood and may provide critical information on the unique nutritional and metabolic needs of rapidly proliferating cancer cells.

Results presented here and our previously published work (Lafita-Navarro et al. 2018) indicate that MYC drives a concomitant increase in Kyn and AHR, suggesting that the Kyn–AHR pathway is activated in MYC transformed cells. Interestingly, only Kyn (the most abundant Trp metabolite present in tumors) and no other Trp metabolites was capable of inducing translocation of AHR into the nucleus of colon cancer cells. Other Trp catabolites downstream from Kyn may perform yet uncharacterized functions in cellular biology and may affect the viability of cancer cells. Indeed, inhibiting Kyn synthesis had more dramatic effects on viability of colon cancer cells than inhibiting the binding of AHR to Kyn. Future investigations targeted at understanding AHR-independent functions of all Kyn catabolites will advance our understanding of the role of Kyn as an oncometabolite.

Previous studies have focused largely on the role of Kyn as a tumor-secreted metabolite that leads to T-cell inactivation, which prevents cancer cell clearance (Opitz et al. 2011; Hughes et al. 2014; Zhai et al. 2015a,b; Liu et al. 2018). Here, however, we demonstrate that Kyn plays a cell-autonomous role in maintaining colon cancer cell proliferation. Indeed, a recent study demonstrated that conditional knockout of the IDO1 gene specifically in colonic cells limits colon cancer development in mice (Bishnupuri et al. 2019).

Oncogenic transformation by MYC (and other oncogenes) often leads to an increase in the uptake of essential amino acids (Yue et al. 2017). However, due to the redundancies in the transporting system and their requirement for normal cell physiology, targeting Trp uptake is not likely to become an exclusive route to specifically eliminate cancer cells. Interestingly, cancer cells displayed an up-regulation of either TDO2 or AFMID (the expression of IDO1 was extremely low in our samples). These results suggest that defining the specific enzymes in the Kyn pathway present in colon cancer patients could inform the choice of inhibitors used to treat these patients and improve the chances of success. Moreover, HPLC-MS methods developed to accurately quantify all stable Trp metabolites in cells and tissues could become the basis to develop a workflow to monitor Kyn and other Trp metabolites during the course of tumorigenesis and to determine whether circulating Trp metabolites might serve as diagnostic or prognostic tools for colon cancer patients.

## Material and methods

### Cell culture

The colonic cell lines DLD1, HT29, HCT116, HCT15, RKO, LoVo (American Type Culture Collection), and HCEC1 CT and the epithelial cell line ARPE-19 were grown in DMEM (4500 mg/L glucose) and sodium bicarbonate without L-glutamine and sodium pyruvate and supplemented with 5% fetal bovine serum. HFFs and *myc*^−/−^ Rat1 fibroblasts and were grown in DMEM supplemented with 10% serum. For the Trp starvation experiments, DMEM F-12 lacking Trp was used (US Biologicals). Recombinant lentiviruses were produced by transfecting HEK293T Phoenix-amphotropic packaging cells with pMD2G (VSV-G protein), pPAX2 (lentivirus packaging vector), and lentiviral constructs using Lipofectamine 3000.

### Cell viability and flow cytometry

For cell viability assays, 10,000 cells were plated in triplicates in 24-well plates prior to siRNA transfection or exposure to pharmacological inhibitors. For knockdown experiments, 40 nM siRNA was used, and the cells were transfected using Lipofectamine RNAi Max. Inhibitors were used at the concentrations indicated in each experiment. For experiments using pharmacological inhibitors, cells were grown in 1% serum medium in the presence of inhibitors or DMSO. After 72 h, the cells were fixed and stained with 0.1% crystal violet dissolved in 20% methanol. The plates were washed three times and destained with 10% acetic acid, and the absorbance of this solution was measured at OD570 to obtain relative viability. For the Trp starvation experiments, 10,000 cells were plated in DMEM supplemented with 5% serum. After 24 h, the medium was replaced by DMEM containing 1% serum with or without 75 µM Trp. The cells were fixed 72 h after replacing the medium and stained as described. Kyn, DMSO, and other metabolites were added directly into the cell culture medium at a concentration of 20 µM for the indicated time points. CH223191 and LM10 were used at 10 µM. The inhibitors 680C91 and epacodostat were used at concentrations ranging from 5 to 100 µM.

For flow cytometry experiments, 1 × 10^5^ cells were grown overnight, and the medium was replaced by DMEM lacking Trp (US Biologicals) or with 75 µM Trp. Cells were cultured for 72 h, and both floating cells and the adherent cells were harvested, fixed in 70% ice-cold ethanol for 2 h at 4°C, and stained with 200 µL of 50 µg/mL stock of PI in the presence of 100 µg/mL RNase. PI emission was measured at 605 nm, and the data were analyzed using FlowJo.

### Growth in soft agar

For soft agar growth, the bottom layer was prepared with 1% agar and mixed (1:1) with DMEM with 10% serum. The top layer was prepared with 0.6% agar and mixed (1:1) with DMEM with 2% serum. Next, 1 × 10^3^ cells were plated in six-well plates in duplicates in DMEM containing 1% serum and the indicated inhibitors. Media were replaced every 4 d, and the colonies were allowed to grow for 4 wk. Colonies were imaged, fixed with 10% formalin, stained with 0.01% crystal violet in 20% methanol, and quantified.

### Organoid culture and growth

Mouse organoids were generated from the following backgrounds: APC^−/−^ (CRISPR), p53^−/−^ (floxed allele, excised), KRas^G12D^ (activated from LSL-KRas^G12D^), and TdTomato red (From Rosa-LSL-TdTomato). AKP organoids were maintained at 37°C as 3D spheroid culture in Matrigel. These were cultured in advanced DMEM/Ham's F-12 supplemented with 1× penicillin/streptomycin and 1×/2 mM glutamax. Minimal basal medium supplemented with 1× B27 and 10 µM Y-27632 was used for Matrigel drops. Organoids were passaged by removing the medium and breaking the Matrigel with PBS. Organoids were then trypsinized for 90 sec at 37°C in a water bath with 1× TrypLE (Express Enzyme; no phenol red), and single cells were pelleted and resuspended in minimal medium (33% of desired plating volume) and Matrigel (Fisher; 66% of desired plating volume). Cells were plated in drops of 10–15 µL.

For organoid formation, AKP organoids were seeded at 13,000 single cells per drop in 12-well plates. Organoids were allowed to grow for 24 h and were incubated with DMSO, 25 µM 680091, and 10 µM CH-223191. Pictures were taken every 24 h and analyzed. Media were changed every 48 h. For 2D growth of the AKP, cells were seeded in six-well cell culture dishes at 50,000 cells per well. Cells were maintained in DMEM high glucose supplemented with 1× penicillin/streptomycin and 1% FBS. For viability experiments, cells were allowed to attach for 6 h after trypsinization and were incubated with DMSO, 25 µM 680091, or 10 µM CH-223191. Pictures were taken every 24 h and analyzed. Plates were stained with crystal violet 2 d after incubation, and relative cell growth was quantified.

### Western blotting, RT-qPCR, and TCGA analyses

Total protein extracts were prepared in RIPA buffer (1× TBS, 1% Nonidet P-40, 0.5% sodium deoxycholate, 0.1% SDS, 0.004% sodium azide with protease inhibitors). Cytoplasmic fractions were isolated using buffer A (10 mM HEPES, 60 mM KCl, 1 mM EDTA, 0.075% [v/v] NP-40 with protease inhibitors). The remaining pellet was washed twice before being resuspended in buffer A and sonicated to yield the nuclear fraction. Protein lysates (20 µg) were processed for Western blotting. RNA was extracted using the RNA extraction kit (Qiagen). Complementary DNA was generated by SuperScript III (Invitrogen) and used with SYBR Green PCR master mix (Applied Biosystem) for qRT-PCR analysis. The mRNA content was normalized to RPS18. All assays were performed using an Applied Biosystems Prism 7900HT sequence detection system. For each mRNA assessment, RT-qPCR analyses were performed at least twice, each time with an *n* = 3. All primers used for RT-qPCR are listed in Supplemental Table S2, and antibodies are listed in Supplemental Table S3.

Normalized gene expression data for colon cancer were downloaded from the TCGA Research Network. Comparison of tumor and normal samples were performed on samples that had matched normal samples. Significance testing between groups was performed using nonparametric Kruskal-Wallis test.

### LC-MS/MS quantification of Trp metabolites

Qualitative assessment of global metabolites by MS (metabolomics) was performed by the Metabolomics Facility at the Children's Research Institute (University of Texas Southwestern) as described previously (Anso et al. 2013; Mullen et al. 2014). Colon cancer tissues were obtained from the University of Texas Southwestern Harold C. Simmons Comprehensive Cancer Center Biorepository Bank. Deidentified samples were obtained under the Institutional Review Board-approved study from the repository center (STU 102010-051). Only patient-matched normal and tumor tissues were used. Time from dissection to freezing was strictly maintained, and cold ischemia time was recorded. Tissues were sonicated and extracted with 80% methanol.

For the cell line studies, the cells were grown in triplicate in 10-cm dishes and, 48 h after plating, were extracted with 1 mL of 80% methanol and spun. The supernatants of the cell lines or tissues were dried using speed vac and were used to measure metabolite concentration. Protein levels were measured using an untreated replica plate for each cell line unless specified in the legend. Dried methanol extractions were dissolved in a resuspension solution consisting of ddH_2_0 containing the internal standard L-Trp-2′,4′,5′,6′,7′-d5 (“d5 Trp” from CDN Isotopes) for 10 min at room temperature, vortexed, and then incubated for 10 min at 37°C. Standards were made by combining equal amounts of untreated samples as a background matrix. The standard mix was diluted with resuspension solution at 1:5000 for Kyn, cinnabarinic acid, and xanthurenic acid standards and 1:20,000 for Trp, kynurenic acid, and serotonin standards. The dilution was optimized to give a signal in blank matrix that was detectable by LC-MS/MS but below the targeted limit of quantitation for each metabolite. A standard curve was prepared by spiking diluted blank matrix with authentic metabolite standards, and the value in blank matrix was then subtracted from each point. The subtracted values were plotted in GraphPad with a quadratic line fit with an *r*^2^ value >0.98. Standard curves contained a minimum of six points (at least two-thirds of total points) that, upon back calculation, were within 15% of theoretical. Triplicate values were inspected for outliers using a Grubbs test (GraphPad). Values were normalized to cellular protein content. Metabolite levels were determined by LC-MS/MS using quantitative MRM methods as developed previously on a Sciex 4000QTRAP or QTRAP6500^+^ mass spectrometer coupled to Shimadzu liquid chromatography systems. MS/MS transitions evaluated were as follows: Kyn, 209.063–94.2; Trp, 205.09–188.0; cinnabarinic acid, 300.99–283; 3-hydroxyanthranillic acid, 153.848–136.1; kynurenic acid, 189.962–144.1; melatonin, 233.023–174.1; serotonin, 176.998–160; xanthurenic acid, 210.206–192.4; and d5-Trp, 210.206–192.40. Samples were separated on a Phenomenex Synergi Polar-RP column (2.0 × 150 mm, 4-µm packing) using reverse phase chromatography with water and acetonitrile, each containing 0.2% acetic acid.

### Measurements of ^13^C-Trp and NAD(H)

Cell lines were grown in 10-cm dishes overnight in triplicate, spiked with 75 µM ^13^C-Trp (Cambridge Isotope Laboratories), and harvested 5 and 30 min after the Trp addition. Cell lysates were then extracted with 0.5 mL of 80% methanol and processed and analyzed as described above. Protein levels were measured for each sample and used to normalize the amounts of each metabolite. ^13^C-Trp was detected with a 216.0–126.0 transition, while ^13^C-Kyn was detected as the 219.1–202.0 transition. Because ^13^C-Kyn did not have an authentic standard, values were reported as Analyte/IS peak area normalized to cellular protein content.

For NAD(H) measurements, 1 × 10^4^ cells were plated per well in a 96-well plate in triplicates. The cells were plated on day 1, and, the next day, inhibitors at the indicated concentrations were added for overnight incubation. Next, 5 mM H_2_O_2_ was added 30 min before lysing the cells. Total NAD(H) measurements were performed as described previously (Sullivan et al. 2015): Briefly, cells were washed three times in cold PBS, extracted in 100 µL of cold lysis buffer (1% dodecyltrimethylammonium bromide [DTAB] in 0.2 N NaOH diluted 1:1 with PBS), and immediately placed on ice. After collection, samples were equilibrated for 10 min to room temperature. Per the manufacturer's instructions, 20 µL of the sample were used to quantitate total NAD(H) using the NAD/NADH-Glo assay kit (Promega, G9071), and luminescence values were normalized by total protein amount. For all experiments, SEM were calculated from triplicate measurements of independent samples for each experimental group, and *P*-values were calculated using Student's *t*-test (two-sided).

### IHC and immunofluorescence

For IHC, 4-mm sections were deparaffinized, rehydrated, and pretreated for 10 min in a microwave (or pressure cooker) in Dako buffer (pH 6; Dako). Samples were then incubated overnight at 4°C with the indicated primary antibodies diluted in RPMI 1640 + 10% bovine serum. Slides were washed twice with TBS/0.1% Tween 20 and then developed with the EnVision system (Dako). Immunofluorescence staining was performed in Labtek 4 chamber slides. Cells were fixed with 4% paraformaldehyde, permeabilized with 0.5% Triton X-100, and blocked with 2% BSA for 1 h at room temperature. Samples were incubated with primary antibody for 1 h at room temperature or overnight at 4°C. Alexa fluor secondary antibody (1:500) was used. DAPI (1 µg/mL) was used to visualize the nucleus. The Kyn antibody was tested for specificity by incubating 1:3 (10 mM Kyn + Kyn antibody) for 30 min before adding to the fixed cells.
